# Biphasic concentration-dependent interaction between imidacloprid and dietary phytochemicals in honey bees (*Apis mellifera*)

**DOI:** 10.1371/journal.pone.0206625

**Published:** 2018-11-01

**Authors:** Michael J. Wong, Ling-Hsiu Liao, May R. Berenbaum

**Affiliations:** Department of Entomology, University of Illinois at Urbana-Champaign, Urbana, Illinois, United States of America; University of California San Diego, UNITED STATES

## Abstract

**Background:**

The presence of the neonicotinoid imidacloprid in nectar, honey, pollen, beebread and beeswax has been implicated in declines worldwide in the health of the western honey bee *Apis mellifera*. Certain phytochemicals, including quercetin and *p*-coumaric acid, are ubiquitous in the honey bee diet and are known to upregulate cytochrome P450 genes encoding enzymes that detoxify insecticides. Thus, the possibility exists that these dietary phytochemicals interact with ingested imidacloprid to ameliorate toxicity by enhancing its detoxification.

**Approach:**

Quercetin and *p-*coumaric acid were incorporated in a phytochemical-free artificial diet individually and together along with imidacloprid at a range of field-realistic concentrations. In acute toxicity bioassays, honey bee 24- and 48- hour imidacloprid LC_50_ values were determined in the presence of the phytochemicals. Additionally, chronic toxicity bioassays were conducted using varying concentrations of imidacloprid in diets with the phytochemicals to test impacts of phytochemicals on longevity.

**Results:**

In acute toxicity bioassays, the phytochemicals had no effect on imidacloprid LC_50_ values. In chronic toxicity longevity bioassays, phytochemicals enhanced honey bee survival at low imidacloprid concentrations (15 and 45 ppb) but had a negative effect at higher concentrations (105 ppb and 135 ppb). *p*-Coumaric acid alone increased honey bee longevity at concentrations of 15 and 45 ppb imidacloprid (hazard ratio (HR): 0.83 and 0.70, respectively). Quercetin alone and in combination with *p*-coumaric acid similarly enhanced longevity at 45 ppb imidacloprid (HR:0.81 and HR:0.77, respectively). However, *p*-coumaric acid in combination with 105 ppb imidacloprid and quercetin in combination with 135 ppb imidacloprid increased honey bee HR by approximately 30% (HR:1.33 and HR:1.30, respectively).

**Conclusions:**

The biphasic concentration-dependent response of honey bees to imidacloprid in the presence of two ubiquitous dietary phytochemicals indicates that there are limits to the protective effects of the natural diet of honey bees against neonicotinoids based on their own inherent toxicity.

## Introduction

The western honey bee (*Apis mellifera* L.) provides important pollination services for major crops around the world [1, 2] and is valued at more than $15 billion per year [3] in the United States alone. However, in recent years losses in overwintering honey bee colonies in the United States have averaged approximately 30% annually [4, 5]. The factors that contribute to colony decline are multifaceted and include parasites, pests, diseases, monoculture agriculture, pesticides, nutrition, and modern beekeeping practices [6]. In particular, neonicotinoid pesticides such as imidacloprid have been a focus of attention [7] because of their systemic activity and widespread use, resulting in consistent presence of residues in nectar and pollen consumed by bees [8], a cause for concern due to their high toxicity to invertebrates [9]. Neonicotinoids are widely used because they are effective against a broad range of crop pests, acting as high-affinity irreversible agonists of the nicotinic acetylcholine receptor. This interaction causes a permanent influx of cations to stimulate surrounding neuronal membranes, where repeated firing of action potentials exhausts cell resources leads to insect paralysis or death [10]. Multiple sub-lethal effects of imidacloprid on honey bees have been documented [11, 12] and include reduced foraging rate [13], decreased mobility[14], decreased learning [15, 16], weakened immunity [17], delayed larval development and reduced adult longevity [18]. These effects have been documented in laboratory experiments at field-realistic doses of less than 20 ppb encompassing studies focused on single acute exposures [15] and chronic exposures [19] and both topical [14] and oral [20] exposure. However, imidacloprid residues vary widely depending on the crop and timing of application and can exceed 20 ppb in field settings [8].

Imidacloprid can disperse throughout the plant because it is a systemic water-soluble pesticide [21]. It exhibits long persistence in the environment [22, 23] and can travel far beyond applied fields, resulting in contamination of other plants via surface and ground water [24]. Honey bees thus encounter imidacloprid via a diversity of routes [23] throughout their life. Foragers can encounter imidacloprid either topically by flying through planter dust or settling on contaminated surfaces, or orally by ingesting nectar, pollen, and water. In water, especially guttation water or morning dew, imidacloprid can be present at even higher concentrations (up to 346 ppm [25, 26]) than in nectar, or pollen (range 0.05–912 ppb [8, 23]). Additionally, honey bees in a hive can encounter imidacloprid residues via ingestion of beebread (up to 912 ppb) [27] and honey (up to 14 ppb) [21] and from contact with beeswax (up to 13.6 ppb) [27], albeit at levels much lower than in agricultural settings [23, 25, 26]. Thus, both acute and chronic toxicity of imidacloprid are important factors to consider when evaluating its effects on honey bees. Although there have been a large number of both laboratory-based and field-based experiments on the acute and chronic toxicity of imidacloprid on honey bees, there exists much debate on the reliability and interpretations of these studies [28–30]. The fact that colony-level variation exists in honey bee sensitivity to imidacloprid [31] means that an extensive sampling effort must be made across multiple colonies when estimating imidacloprid toxicity. Seasonality [32], nutritional [33], environmental, and endogenous factors [34] all have the potential to induce variation in responses to neonicotinoid toxicity.

Honey bees typically ingest neonicotinoids as a contaminant in nectar, honey, pollen, and beebread, all of which are rich in phytochemicals. Honey bees thus of necessity consume neonicotinoids in a matrix of phytochemicals. They rely primarily on cytochrome P450 monooxygenases in the CYP6 and CYP9 families for detoxification of both phytochemicals and pesticides, including neonicotinoids [35, 36]. Exposure to sublethal levels of imidacloprid [37, 38] or dietary phytochemicals [39–43] results in upregulation of CYP6 and CYP9 P450s. In the specific case of the phenolic acid *p-*coumaric acid and the flavonol quercetin, found ubiquitously in honey, pollen and propolis, members of the CYP6AS subfamily and the CYP9Q subfamily are upregulated in both adults and larvae [41–43]. Whereas the CYP6AS subfamily is associated primarily with flavonoid metabolism [44, 45], enzymes in the CYP9Q subfamily metabolize both phytochemicals and pesticides, including imidacloprid in the case of CYP9Q1, CYP9Q2, and CYP9Q3 [36].

Accordingly, the possibility exists that co-occurring dietary phytochemicals alter behavioral or physiological responses of honey bees to imidacloprid. Quercetin reduces the acute toxicity of the pyrethroid tau-fluvalinate [39], and both quercetin and *p-*coumaric acid increase longevity of honey bees in the presence of two pyrethroids, β-cyfluthrin and bifenthrin [46]. All three of these pyrethroid insecticides are metabolized by members of the CYP9Q subfamily, suggesting that amelioration of pesticide toxicity by *p-*coumaric acid and quercetin is due to induction of these enzymes. A similar interaction might also exist between imidacloprid and phytochemicals because it is also metabolized by members of the CYP9Q subfamily [36] and processed by efflux transporters [47]. Other possible mechanisms of interaction are suggested by the findings of Guseman et al. [48], who showed that quercetin synergizes the toxicity of ivermectin and the neonicotinoid acetamiprid, possibly via interference with transporter proteins within the ABCB, ABCC and ABCG subfamilies.

In view of evidence that *p-*coumaric acid and quercetin upregulate P450 genes encoding pesticide-metabolizing enzymes, their consumption may affect the LC_50_ and lifespan of honey bees exposed to imidacloprid. In this study, we assessed the acute and chronic toxicological effects of imidacloprid in the presence of these two phytochemicals. To assess phytochemical effects on acute imidacloprid toxicity, LC_50_ values for imidacloprid were determined at 24- and 48 hours with the addition of quercetin and *p-*coumaric acid individually or in combination. Additionally, to assess the effects of phytochemicals on chronic imidacloprid toxicity, bees were challenged with a range of field-realistic concentrations of imidacloprid in a longevity bioassay with diets containing quercetin, *p-*coumaric acid, or both phytochemicals.

## Materials and methods

### Honey bees and general experimental procedure

Three *A*. *mellifera* colonies located in an apiary maintained by the University of Illinois at Urbana-Champaign in Urbana, IL were utilized during the summer of 2017. The colonies showed no signs of disease during the experimental period, and varroa mite counts were kept low through the end of summer (0 mite/300 bees for all colonies at the beginning of summer and 0, 9, 16 mites/300 bees at the end of summer) with the monitoring method of the standard alcohol wash procedure [49]. Frames of capped brood were taken from each colony and then moved into a dark incubator kept at 34°C and 50% humidity. After emergence within the incubator, the one-day-old bees were collected every 24 hours from frames until there were sufficient numbers of cohort bees from the same day and colony for at least one replicate.

Once a sufficient number of one-day-old bees emerged from the frames, for both the LC_50_ and longevity assay, they were transferred into closed clear 266-mL plastic cups with multiple ventilation holes and two larger holes for the insertion of food and water feeder tubes. The general arrangement and positioning of experimental cages and feeders were modified from our previous research [46]. For the duration of the experiment, caged bees were kept in a dark incubator room at 34°C and 50% humidity. Each cage contained 25 newly-emerged day-old bees, as well as one-tenth of an artificial queen mandibular gland pheromone strip (DC-715, Mann Lake Ltd., Hackensack, MN) to simulate the pheromonal attributes of a queenright colony [46]. The cages were immediately provisioned with the appropriate treatment diet and deionized water in two 2-mL microcentrifuge feeder tubes with a 6-mm opening on the top for the bees to access *ad libitum*.

### Chemicals and initial treatment preparation

Imidacloprid (PS2086), quercetin (Q4951), *p-*coumaric acid (C9008), and casein (C3400) were purchased from Sigma–Aldrich Co. LLC. (St. Louis, MO), and were used to create treatment diets for the bioassays. Dimethyl sulfoxide (DMSO; D128, Fisher Scientific International, Inc., Pittsburgh, PA) was used as a solvent. Imidacloprid and the two phytochemicals were first dissolved in DMSO to make the 400× or higher concentrated stock solutions, which were then stored at -20°C.

A 50% (w/v) sucrose water diet with casein was prepared at a ratio of 1:12 protein to carbohydrate [46, 50] as the base diet. The base diet was made before use and stored at 4°C for no longer than a week. Casein was used as the primary source of protein because of its phytochemical-free nature and its long-established use as a protein source in insect artificial diets [51], including honey bee diets [46, 50]. Stock solutions of imidacloprid and phytochemicals were then added to prepare all diets freshly for use in both short-term LC_50_ and long-term longevity assays. The base diet was augmented with phytochemical and/or imidacloprid DMSO stock solutions to freshly prepare different treatment diets for each assay. For the control diet, DMSO was also added to the base diet. As the result, the final concentration of all diets was 0.25% (v/v) DMSO.

### Effects of phytochemicals on honey bee imidacloprid LC_50_ values for honey bees

For LC_50_ assays, all quercetin-containing treatment diets were made up at a final concentration of 0.25 mM, and all *p-*coumaric acid-containing treatment diets were made up at a final concentration of 0.5 mM. These concentrations were chosen based on their potential for biological activity [42, 43, 46] and their activity in feeding preference behavior [52]; moreover, these represent concentrations well within the range typically encountered by adult honey bees [53–55]. Imidacloprid-containing treatment diets were prepared at concentrations of 0, 5, 10, 15, 20, and 25 ppm; these concentrations fall within the range encountered by bees under field conditions, (as in guttation water [25, 26]). Diets were replaced every 24 hours for each cage.

Honey bees were checked for mortality every 24 hours over a 48-hour time period. Food consumption data were not recorded but were assumed to be approximately 106.04–568.19 ng/bee/day of imidacloprid consumed based on the consumption rates measured in a similar study [46]. Bees that were immobile and unable to right themselves were scored as dead. Three replicates of each treatment from each of three colonies were used for this experiment, for a total of 9 replicates of 24 treatments (4 phytochemical treatments plus 6 imidacloprid concentrations) and resulted in a total of 5,400 honey bees at 216 cages in this LC_50_ bioassay. All replicate treatments from a single colony were carried out on the same day, and all three colonies were tested within the same month (July 2017).

### Effects of phytochemicals and imidacloprid on honey bee longevity

Diets were prepared for the longevity assays by the same methods used to prepare diets for the LC_50_ assay. All treatments containing quercetin or/and *p-*coumaric acid were prepared at final concentrations of 0.25 mM and 0.5 mM, respectively. Treatments containing imidacloprid were prepared at concentrations of 0, 15, 45, 75, 105, and 135 ppb. These values represent the low range of field-realistic concentrations of imidacloprid in agricultural settings [8, 21, 23, 27] (in order to capture chronic rather than acute responses). Based on a similar longevity study [46], we estimate that 0.32–3.07 ng/bee/day of imidacloprid were consumed in this study. Diets were replaced every day. Water tubes were replaced every five days or when the volume of water fell below 1 mL. Cages were checked for mortality daily until the death of the last bee in each treatment. Overall, there were a total of 24 treatments with and without quercetin and/or *p-*coumaric acid with a range of imidacloprid concentrations. Three replicates of each treatment from each of the three colonies were used for this experiment, for a total of 216 cages and 5,400 honey bees in this longevity bioassay. Every treatment in each replicate began on the same day, with all replicate treatments from a single colony initiated within a three-day interval. All three colonies were tested with three weeks apart between June 2017 and August 2017

### Statistical analysis

Statistical analyses were conducted using R version 3.1.1 (R Core Team, Vienna, Austria) and SPSS software (version 22.0; IBM Corp., Armonk, NY, USA). OriginPro 2016 software (OriginLab Corporation, Northampton, MA, USA) was used to plot Kaplan-Meier survival curves. For the LC_50_ bioassays, Probit analysis was conducted using the “survival” statistical package to estimate the median lethal concentration needed to kill 50% of the honey bees over each 24-hour interval over 48 hours. The 95% confidence intervals for the corresponding LC_50_ values were determined using Fieller’s method [56]. Comparisons across treatments and colonies were made by analyzing the data for overlapping confidence intervals, in addition to performing pairwise LC_50_ likelihood ratio tests [57]. The pairwise ratio comparisons were considered significant if their 95% confidence interval included a “1” between the upper and lower bounds.

For the longevity bioassays, Cox's proportional hazards regression models [58] were used to evaluate the hazard of death according to dietary phytochemicals and imidacloprid concentrations, adjusting for the hive identity as a covariate stratum. By this regression model, hazard ratio (HR) provides an estimate of effect size [59]. The relationship between the HR and survival functions can be characterized as S_*T*_(*t*) = S_*C*_(*t*)^HR^, where S_*T*_(*t*) and S_*C*_(*t*) are the survival probability of treatment and control group at time *t*, respectively. When an effect of treatment was significant in the Cox model, the HR was calculated to express the magnitude of the effect of treatment. When HR > 1, the treatment factor presents a higher risk than that of the control group, and, when HR < 1, the treatment factor reduces the hazard risk relative to that of the control group. The Kaplan-Meier estimator was used to plot the survival curves and estimated mean and median. Of available tests for differences between survival curves, the log-rank test was used (treatment vs. control) to determine significance because of its power and its frequent use in survival analyses.

## Results

### Effects of phytochemicals on imidacloprid LC_50_ values for honey bees

Results from the analysis of overlapping confidence intervals and the pairwise likelihood ratio tests showed that there were no significant differences between LC_50_ values for imidacloprid after 24 hours on the different phytochemical treatment diets (Table 1). All 24-hour imidacloprid LC_50_ values ranged between 10.7 ppm– 11.3 ppm imidacloprid across the phytochemical treatments. Similarly, no significant differences in imidacloprid LC_50_ values were found between treatments after 48 hours (Table 1). No overlapping confidence intervals were found between the treatments, and likelihood ratio tests also resulted in no significant differences. All 48-hour imidacloprid LC_50_ values ranged from 5.8 ppm– 6.8 ppm imidacloprid across the phytochemical treatments.

**Table 1:** Median-lethal concentration (LC_50_) for imidacloprid on diets varying in phytochemical content after 24 and 48 hours.

Phytochemical	n	24-hour		48-hour	
		LC_50_ (ppm)	95% CI^a^ (ppm)	LC_50_ (ppm)	95% CI (ppm)
phytochemical-free	1350	11.18	9.48–12.88	6.83	5.12–8.28
0.25mM p-coumaric acid	1350	11.27	9.81–12.74	6.25	4.976–7.35
0.5mM quercetin	1350	10.70	9.41–11.95	5.83	4.25–7.14
quercetin + *p*-coumaric acid	1350	11.19	9.77–12.60	6.28	5.363–7.11

Bees were provided with five concentrations of imidacloprid-containing treatment diets (5, 10, 15, 20, and 25 ppm) and a DMSO control. n = a total number of bees included in the bioassay, LC_50_ = lethal concentration 50% calculated by the probit model

^a^ CI: Confidence Interval, confidence interval calculated using Fieller’s method

### Effects of phytochemicals and imidacloprid on honey bee longevity

In chronic toxicity longevity bioassays, the Cox proportional hazards models (Cox model) analysis on the pooled results of 5,400 caged honey bees revealed that the hive source (p<0.001) affected the longevity of adult bees. This finding indicates that there is colony-level variation in phytochemical and imidacloprid sensitivity in the tested honey bee colonies (S1 Table). Thus, we used the hive identity as an adjusted stratum in the rest of our analysis.

Across all concentrations of imidacloprid treatments in the longevity assay, the presence of imidacloprid slightly reduced the average longevity of bees (HR: 1.001, p<0.001), while the presence of phytochemicals had no effect. However, within each concentration of imidacloprid, the phytochemicals had a concentration-dependent effect (Table 2). In the absence of imidacloprid, both phytochemicals individually, but not in combination, enhanced longevity and reduced the hazard ratio by approximately 19% (HR: 0.81) compared to diets lacking phytochemicals. Additionally, the two phytochemicals in combination had a negative effect (HR: 1.26) in which there was greater mortality risk compared to *p*-coumaric acid or quercetin alone in the absence of pesticides. In diets with low imidacloprid concentrations, *p*-coumaric acid increased longevity at 15 ppb (HR: 0.83), whereas *p-*coumaric acid and quercetin, both individually and in combination, increased longevity at 45 ppb (HR: 0.70–0.81), (Table 2). At 75 ppb, imidacloprid, the two phytochemicals did not affect lifespan. However, in diets with higher imidacloprid concentrations, the presence of *p*-coumaric acid reduced longevity at 105 ppb (HR: 1.33) and the presence of quercetin reduced longevity at 135 ppb (HR: 1.30) when compared to the phytochemical-free control treatment.

**Table 2:** Cox proportional hazards model analysis of effects of dietary phytochemicals in the presence of imidacloprid on adult bee longevity.

imidacloprid	phytochemical	Estimate	Standard error	*χ*^2^	*df*	*P*	Hazard ratio
0 ppb	phytochemical-free			28.88	3	0.000***	
	*p*-coumaric acid	-0.22	0.10	5.02	1	0.025*	0.81
	quercetin	-0.21	0.10	4.67	1	0.031*	0.81
	quercetin + *p*-coumaric acid	0.23	0.10	5.81	1	0.016*	1.26
15 ppb	control			4.51	3	0.211	
	*p*-coumaric acid	-0.19	0.10	3.85	1	0.050*	0.83
	quercetin	-0.16	0.10	2.73	1	0.099	0.85
	quercetin + *p*-coumaric acid	-0.10	0.10	1.08	1	0.299	0.91
45 ppb	phytochemical-free			14.93	3	0.002**	
	*p*-coumaric acid	-0.36	0.10	14.21	1	0.000***	0.70
	quercetin	-0.21	0.10	4.89	1	0.027*	0.81
	quercetin + *p*-coumaric acid	-0.26	0.10	7.15	1	0.008**	0.77
75 ppb	phytochemical-free			3.90	3	0.273	
	*p*-coumaric acid	0.02	0.10	0.06	1	0.814	1.02
	quercetin	0.00	0.10	0.00	1	0.973	1.00
	quercetin + *p*-coumaric acid	-0.15	0.10	2.36	1	0.124	0.86
105 ppb	phytochemical-free			9.13	3	0.028*	
	*p*-coumaric acid	0.29	0.10	8.84	1	0.003**	1.33
	quercetin	0.10	0.09	1.04	1	0.309	1.10
	quercetin + *p*-coumaric acid	0.13	0.10	1.84	1	0.175	1.14
135 ppb	phytochemical-free			9.55	3	0.023*	
	*p*-coumaric acid	0.03	0.10	0.11	1	0.737	1.03
	quercetin	0.26	0.10	7.56	1	0.006**	1.30
	quercetin + *p*-coumaric acid	0.15	0.10	2.54	1	0.111	1.17

When hazard ratio > 1, the treatment factor presents a higher risk than that of the phytochemical-free group, and, when hazard ratio < 1, the treatment factor reduces the hazard risk than that of the phytochemical-free group.

* *p* < 0.05

** *p* < 0.01

*** *p* < 0.001.

**Fig 1:**
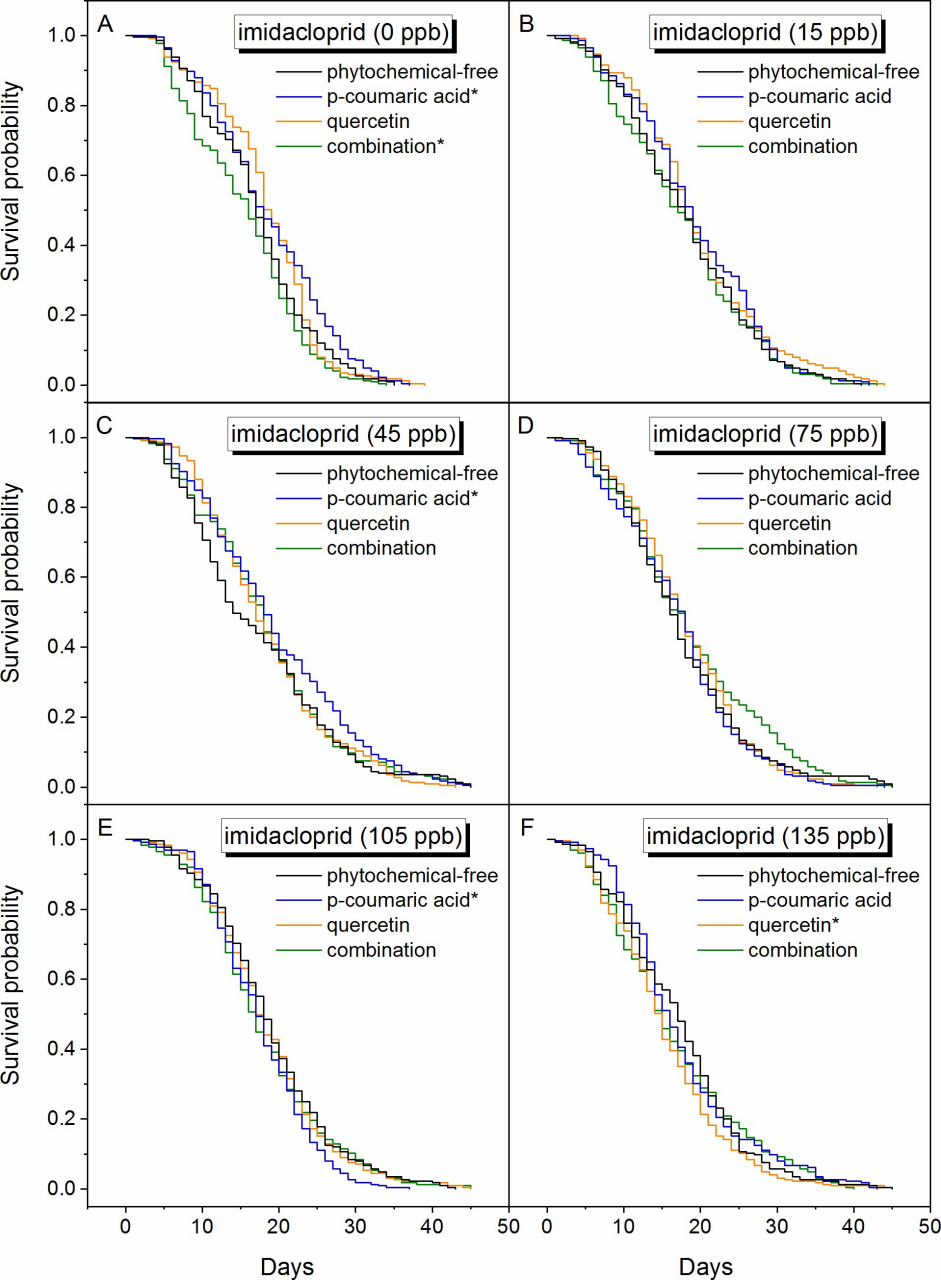
Kaplan–Meier plot of honey bee survival function on different concentrations of imidacloprid with different phytochemical supplements. These diets were (A) imidacloprid-free (B) 15 ppb imidacloprid, (C)45 ppb imidacloprid, (D) 75 ppb imidacloprid, (E) 105 ppb imidacloprid, and (F) 1355 ppb imidacloprid. (Total 5,400 bees were tested; *n* = 225 for each phytochemical sub-group; Log-rank test between treatments and control, * *p* < 0.05).

Within the Kaplan-Meier survivorship curves (Fig 1), differences in survival rates first appear to emerge between the fifth and tenth day within each treatment. Honey bees consuming *p*-coumaric acid experienced increased longevity compared to honey bees consuming the control diet in the absence of imidacloprid (log-rank test, χ^2^ = 6.52, p = 0.01; S2 and S3 Tables), whereas honey bees consuming *p*-coumaric acid and quercetin together had decreased longevity compared to bees consuming the control diet (χ^2^ = 5.40, p = 0.02), (Fig 1A). At 45 ppb imidacloprid, honey bees consuming *p*-coumaric acid had greater longevity than those feeding on the control diet (χ^2^ = 5.19, p = 0.02), (Fig 1C). At 75 ppb imidacloprid, there were no significant differences between the different phytochemical treatments (Fig 1D). At 105 ppb imidacloprid, honey bees consuming *p-*coumaric acid had decreased longevity compared to bees consuming the control diet (χ^2^ = 658, p = 0.01), (Fig 1E), while at 135 ppb imidacloprid bees consuming quercetin experienced decreased longevity compared to bees consuming the control diet (χ^2^ = 5.09, p = 0.02), (Fig 1F).

## Discussion

The phytochemicals *p-*coumaric acid and quercetin have the potential to increase honey bee longevity in the absence of pesticides. They can also prolong survival during exposure to imidacloprid, a phenomenon previously documented with exposure to pyrethroids [39, 46]. This study both expands the inventory of examples of phytochemical interaction with pesticide toxicity and provides insight into the mechanisms underlying these interactions. The results of the chronic exposure bioassay showed a biphasic concentration-dependent response in bees, according to which *p*-coumaric acid and quercetin are beneficial to bees ingesting imidacloprid at low concentrations (45 ppb), but detrimental when concentrations of imidacloprid reach higher levels (105 and 135 ppb). At an intermediate level (75 ppb imidacloprid), phytochemical effects were undetectable. Additionally, in the absence of phytochemicals, sublethal concentrations of imidacloprid did not alter honey bee longevity (S4 Table). The biphasic response we found suggests that a hormetic interaction exists [60–63] between imidacloprid and phytochemicals on honey bee survival according to which low concentrations of synthetic xenobiotics in combination with phytochemicals result in higher viability, but higher concentrations of synthetic xenobiotics in combination with the same phytochemicals become toxic. This hormetic response has been demonstrated for other pesticides in honey bees [64, 65].

The benefits of consuming phytochemicals found in honey, pollen, and beebread for honey bees at low levels of imidacloprid may stem from their capacity to upregulate detoxifying P450s [39–41, 66]. Mao et al. [40–43] showed that phytochemicals, including *p-*coumaric acid and quercetin, differentially upregulate genes in the CYP6AS and CYP9Q subfamilies. Although the P450 inhibitors piperonyl butoxide, triflumizole, and propiconazole do not increase the toxicity of imidacloprid to honey bees [67], Manjon et al. [36] conclusively demonstrated that another P450 inhibitor, 1-aminobenzotriazole, increased sensitivity to imidacloprid by 2.7-fold and at the same time demonstrated via radioligand binding and functional expression studies that CYP9Q enzymes determine bee sensitivity to neonicotinoids. Thus, upregulation of relevant pesticide-detoxifying P450s by phytochemicals likely accounts for the enhanced longevity of bees consuming low levels of imidacloprid.

At concentrations of imidacloprid at or greater than 105 ppm, the presence of phytochemicals decreases longevity. One possible explanation may be that bees appear to rely on a very small number of broadly substrate-specific CYP9 enzymes to cope with neonicotinoids and other pesticides [36, 40]. In view of the fact that CYP9 enzymes also contribute to phytochemical metabolism, competition for access to the enzyme catalytic sites may reduce the beneficial effects of phytochemicals, which themselves have toxic effects when P450 activity is inhibited [40]. As a result, quercetin, imidacloprid, or both may accumulate and result in decreased lifespan. Additionally, higher concentrations of imidacloprid likely induce oxidative stress in insects, as they do in vertebrates (e.g. [68–70]). Given that neonicotinoids have a much higher selectivity factor for nicotinic acetylcholine receptors in insects compared to vertebrates [71], the resulting oxidative stress is also likely to be much greater. Additionally, imidacloprid downregulates both antioxidant and immunity genes in honey bee queens [72]; protective effects of both *p-*coumaric acid and quercetin against imidacloprid may result from their antioxidant properties, reducing pesticide-induced oxidative stress [73–76] at low levels of imidacloprid. Higher concentrations of imidacloprid with the concomitant oxidative stress associated with its toxicity may overwhelm the protective antioxidative effects of these two phytochemicals.

In our acute LC_50_ toxicity tests of imidacloprid, consuming the phytochemicals individually or in combination all failed to provide beneficial effects. Thus, it appears that protective effects of phytochemicals against imidacloprid build up over a longer period of time than within the 24–48 hour timeframe used here for acute toxicity bioassays. However, this is not necessarily the case for all pesticides encountered by honey bees; quercetin decreased the toxic effects of tau-fluvalinate within 24 hours [39]. A possible explanation for the failure of phytochemicals to “rescue” bees from toxic effects of imidacloprid could relate to the fact that the toxicity of imidacloprid manifests itself through a delayed reaction compared to other pesticides [20]. In addition, even though imidacloprid is metabolized rapidly in honey bees (five hour elimination half-life), its metabolites persist longer and are more toxic than imidacloprid[77]. Thus, detection of amelioration of toxicity over a 24- to 48-hour assay is unlikely. The fact that the 48-hour LC_50_ values for all treatments were consistently greater than the 24-hour LC50 values is likely attributable to the delayed toxic effects of imidacloprid compared to other pesticides [20]. Moreover, a cumulative toxic effect may have been involved. In our earlier study with tau-fluvalinate [39], the bees were challenged only at a single timepoint with the pesticide. In our current study, tested bees consumed imidacloprid + sugar water during the entire experimental period, which might negate the phytochemical “rescue” effect.

The LC_50_ of imidacloprid calculated in this study is considerably greater than values reported in other studies. Although LC_50_ values ranging from 54 ppb to as high as 600 ppb after 48 hours [20] were determined in other studies for honey bees, this study yielded an LC_50_ value approximating 6 ppm, an order of magnitude higher than previously reported values. Discrepancies in these values may be attributable to colony-level differences in imidacloprid toxicity, as there does appear to be a strong colony effect regarding imidacloprid sensitivity [31, 33]. This possibility is further supported by the fact that many studies have estimated LC_50_ values for imidacloprid in honey bees that vary by a factor of more than 100-fold (e.g. [20, 78]). This inter-colony variation in imidacloprid toxicity across different studies may reflect many factors, including differences in colony strength, disease presence, and colony food stores [31, 33, 34].

Quercetin and *p-*coumaric acid represent only two of a diversity of phytochemicals encountered by honey bees even over the course of a season in a single locality. The phytochemical composition of honey varies with the identity of plants from which nectar was collected to make the honey and consequently most honeys contain multiple phenolic acids and flavonols [79] as well as other constituents, including terpenoids, sulfur-containing compounds, and alkaloids [80, 81]. Thus, at the same time bees ingest a diversity of pesticides due to hive contamination [27], they also ingest complex mixtures of phytochemicals that may collectively have greater protective effects than have been detected here with just one phenolic acid (*p-*coumaric acid) and one flavonol (quercetin) [41], or, alternatively, greater antagonistic effects due to competitive inhibition of detoxification enzyme activity. Such interactions, particularly when multiple pesticides are involved, may have critical impacts on bee health in agricultural environments [67, 82], remain poorly understood. The biphasic concentration-dependent response of honey bees to imidacloprid in the presence of two ubiquitous dietary phytochemicals indicates that there are limits to the protective effects of the natural diet of honey bees against neonicotinoids based on their own inherent toxicity. At the same time, it serves as a reminder that the benefits of a phytochemically diverse diet consumed by bees may be altered in contemporary landscapes exposing them to a diet diverse in insecticides and other pesticides.

## Supporting information

S1 Table Summary hazard ratios of adult bee longevity from individual and combination data of each hive by effect of dietary phytochemicals and imidacloprid, obtained from Cox proportional hazards model analysis. (DOCX)

S2 Table Means and medians for survival time. (DOCX)

S3 Table Summary of the log-rank test between phytochemical-free group and each phytochemical treatment in different imidacloprid concentration. (DOCX)

S4 Table Cox proportional hazards model analysis of effects of imidacloprid in different concentration with a phytochemical-free diet on adult bee longevity. (DOCX)
