# Supplementary material for: Biphasic concentration-dependent interaction between imidacloprid and dietary phytochemicals in honey bees (Apis mellifera)
Source: PLoS One. 2018 Nov 1;13(11):e0206625. doi: 10.1371/journal.pone.0206625 (PMC6211726; doi:10.1371/journal.pone.0206625)
Supplement: S1 Table — (DOCX) [file pone.0206625.s001.docx]

**S1 Table. Summary hazard ratios of adult bee longevity from individual and combination data of each hive by effect of dietary phytochemicals and imidacloprid, obtained from Cox proportional hazards model analysis**

| imidacloprid (ppb) | Phytochemical | *df* | hive Y24 | | hive Y25 | | hive W61 | |
| --- | --- | --- | --- | --- | --- | --- | --- | --- |
|  |  |  | Sig. | Hazard ratio | Sig. | Hazard ratio | Sig. | Hazard ratio |
| 0 | CD ^a^ | 3 | 0.00*** |  | 0.04* |  | 0.00*** |  |
|  | Pc | 1 | 0.17 | 1.26 | 0.68 | 0.94 | 0.00*** | 0.47 |
|  | Qc | 1 | 0.01** | 0.63 | 0.63 | 0.92 | 0.96 | 0.99 |
|  | QP | 1 | 0.02* | 1.46 | 0.04* | 1.41 | 0.76 | 0.95 |
| 15 | CD | 3 | 0.61 |  | 0.01** |  | 0.00*** |  |
|  | Pc | 1 | 0.50 | 0.90 | 0.57 | 1.10 | 0.00** | 0.59 |
|  | Qc | 1 | 0.25 | 0.83 | 0.05 | 0.73 | 0.50 | 1.12 |
|  | QP | 1 | 0.24 | 0.82 | 0.19 | 1.24 | 0.05 | 0.72 |
| 45 | CD | 3 | 0.00*** |  | 0.08 |  | 0.87 |  |
|  | Pc | 1 | 0.00*** | 0.39 | 0.23 | 0.82 | 0.45 | 0.88 |
|  | Qc | 1 | 0.00*** | 0.42 | 0.22 | 1.23 | 0.50 | 0.89 |
|  | QP | 1 | 0.00*** | 0.37 | 0.45 | 1.13 | 0.70 | 0.94 |
| 75 | CD | 3 | 0.89 |  | 0.12 |  | 0.36 |  |
|  | Pc | 1 | 0.48 | 0.89 | 0.45 | 1.13 | 0.69 | 1.07 |
|  | Qc | 1 | 0.54 | 0.90 | 0.63 | 0.92 | 0.27 | 1.20 |
|  | QP | 1 | 0.75 | 0.95 | 0.11 | 0.77 | 0.51 | 0.90 |
| 105 | CD | 3 | 0.07 |  | 0.01* |  | 0.81 |  |
|  | Pc | 1 | 0.01* | 1.51 | 0.00** | 1.68 | 0.61 | 0.92 |
|  | Qc | 1 | 0.07 | 1.34 | 0.47 | 1.13 | 0.35 | 0.86 |
|  | QP | 1 | 0.06 | 1.37 | 0.34 | 1.17 | 0.46 | 0.89 |
| 135 | CD | 3 | 0.00*** |  | 0.42 |  | 0.12 |  |
|  | Pc | 1 | 0.35 | 1.17 | 0.61 | 0.92 | 0.94 | 1.01 |
|  | Qc | 1 | 0.03* | 1.43 | 0.29 | 1.19 | 0.11 | 1.30 |
|  | QP | 1 | 0.00*** | 2.06 | 0.77 | 0.95 | 0.42 | 0.88 |

^a^ CD, diet lacking phytochemicals; Pc, diet containing 0.5 mM *p*-coumaric acid; Qc, diet containing 0.25 mM quercetin; QP, diet containing 0.25 mM quercetin and 0.5 mM *p*-coumaric acid. (Total 5,400 bees were tested; *n* = 225 for each phytochemical sub-group. * *p* < 0.05; ** *p* < 0.01; *** *p* < 0.001. )
