# Supplementary material for: Biphasic concentration-dependent interaction between imidacloprid and dietary phytochemicals in honey bees (Apis mellifera)
Source: PLoS One. 2018 Nov 1;13(11):e0206625. doi: 10.1371/journal.pone.0206625 (PMC6211726; doi:10.1371/journal.pone.0206625)
Supplement: S2 Table — (DOCX) [file pone.0206625.s002.docx]

**S2 Table. Means and medians for survival time**

| imidacloprid (ppb) | Phytochemical | n | Mean | | | | Median | | | |
| --- | --- | --- | --- | --- | --- | --- | --- | --- | --- | --- |
|  |  |  | days | SE | 95% Confidence Interval | | days | SE | 95% Confidence Interval | |
|  |  |  |  |  | Lower Bound | Upper Bound |  |  | Lower Bound | Upper Bound |
| 0 | CD^a^ | 225 | 17.1 | 0.5 | 16.2 | 18.0 | 17.0 | 0.5 | 16.0 | 18.0 |
|  | Pc | 225 | 18.6 | 0.5 | 17.6 | 19.6 | 18.0 | 0.9 | 16.3 | 19.7 |
|  | Qc | 225 | 18.2 | 0.5 | 17.3 | 19.1 | 19.0 | 0.5 | 18.0 | 20.0 |
|  | QP | 225 | 15.3 | 0.5 | 14.4 | 16.3 | 16.0 | 0.8 | 14.5 | 17.5 |
|  | Overall | 900 | 17.3 | 0.2 | 16.8 | 17.8 | 18.0 | 0.3 | 17.5 | 18.5 |
| 15 | CD | 225 | 18.1 | 0.5 | 17.0 | 19.2 | 18.0 | 0.7 | 16.7 | 19.3 |
|  | Pc | 225 | 19.2 | 0.6 | 18.1 | 20.3 | 19.0 | 0.6 | 17.7 | 20.3 |
|  | Qc | 225 | 19.4 | 0.6 | 18.3 | 20.5 | 18.0 | 0.5 | 17.0 | 19.0 |
|  | QP | 225 | 17.5 | 0.6 | 16.3 | 18.6 | 17.0 | 0.9 | 15.3 | 18.7 |
|  | Overall | 900 | 18.5 | 0.3 | 18.0 | 19.1 | 18.0 | 0.3 | 17.3 | 18.7 |
| 45 | CD | 225 | 17.0 | 0.6 | 15.8 | 18.2 | 14.0 | 1.1 | 11.8 | 16.2 |
|  | Pc | 225 | 19.5 | 0.6 | 18.2 | 20.7 | 18.0 | 0.7 | 16.6 | 19.4 |
|  | Qc | 225 | 18.2 | 0.5 | 17.2 | 19.3 | 17.0 | 0.7 | 15.6 | 18.4 |
|  | QP | 225 | 18.0 | 0.6 | 16.9 | 19.2 | 18.0 | 0.7 | 16.7 | 19.3 |
|  | Overall | 900 | 18.2 | 0.3 | 17.6 | 18.8 | 17.0 | 0.4 | 16.2 | 17.8 |
| 75 | CD | 225 | 17.4 | 0.6 | 16.3 | 18.5 | 16.0 | 0.6 | 14.8 | 17.2 |
|  | Pc | 225 | 16.9 | 0.5 | 15.9 | 18.0 | 18.0 | 0.7 | 16.7 | 19.3 |
|  | Qc | 225 | 17.9 | 0.5 | 16.9 | 18.9 | 18.0 | 0.6 | 16.9 | 19.1 |
|  | QP | 225 | 18.6 | 0.6 | 17.4 | 19.8 | 17.0 | 0.9 | 15.3 | 18.7 |
|  | Overall | 900 | 17.7 | 0.3 | 17.2 | 18.2 | 17.0 | 0.3 | 16.3 | 17.7 |
| 105 | CD | 225 | 18.8 | 0.5 | 17.8 | 19.8 | 18.0 | 0.5 | 17.0 | 19.0 |
|  | Pc | 225 | 17.5 | 0.4 | 16.6 | 18.3 | 17.0 | 0.5 | 16.1 | 17.9 |
|  | Qc | 225 | 18.5 | 0.5 | 17.5 | 19.5 | 17.0 | 0.5 | 16.1 | 17.9 |
|  | QP | 225 | 17.9 | 0.5 | 16.8 | 18.9 | 17.0 | 0.6 | 15.9 | 18.1 |
|  | Overall | 900 | 18.2 | 0.3 | 17.7 | 18.7 | 17.0 | 0.3 | 16.5 | 17.5 |
| 135 | CD | 225 | 17.0 | 0.5 | 16.0 | 18.1 | 17.0 | 0.7 | 15.7 | 18.3 |
|  | Pc | 225 | 17.3 | 0.5 | 16.3 | 18.4 | 16.0 | 0.7 | 14.7 | 17.3 |
|  | Qc | 225 | 15.3 | 0.5 | 14.3 | 16.3 | 15.0 | 0.5 | 14.1 | 15.9 |
|  | QP | 225 | 16.4 | 0.6 | 15.2 | 17.5 | 15.0 | 0.7 | 13.6 | 16.4 |
|  | Overall | 900 | 16.5 | 0.3 | 16.0 | 17.1 | 15.0 | 0.3 | 14.3 | 15.7 |
| Overall | Overall | 5400 | 17.7 | 0.1 | 17.5 | 18.0 | 17.0 | 0.1 | 16.7 | 17.3 |

^a^ CD, diet lacking phytochemicals; Pc, diet containing 0.5 mM *p*-coumaric acid; Qc, diet containing 0.25 mM quercetin; QP, diet containing 0.25 mM quercetin and 0.5 mM *p*-coumaric acid.
