# Supplementary material for: Biphasic concentration-dependent interaction between imidacloprid and dietary phytochemicals in honey bees (Apis mellifera)
Source: PLoS One. 2018 Nov 1;13(11):e0206625. doi: 10.1371/journal.pone.0206625 (PMC6211726; doi:10.1371/journal.pone.0206625)
Supplement: S3 Table — (DOCX) [file pone.0206625.s003.docx]

**S3 Table. Summary of the log-rank test between phytochemical-free group and each phytochemical treatment in different imidacloprid concentration.**

| imidacloprid (ppb) | Phytochemical | Chi-Square | Sig. |
| --- | --- | --- | --- |
| 0 | Pc ^a^ | 6.52 | 0.01* |
|  | Qc | 2.25 | 0.13 |
|  | QP | 5.40 | 0.02* |
| 15 | Pc | 2.08 | 0.15 |
|  | Qc | 2.52 | 0.11 |
|  | QP | 0.16 | 0.69 |
| 45 | Pc | 5.19 | 0.02* |
|  | Qc | 0.40 | 0.53 |
|  | QP | 0.23 | 0.63 |
| 75 | Pc | 0.23 | 0.63 |
|  | Qc | 0.12 | 0.72 |
|  | QP | 2.52 | 0.11 |
| 105 | Pc | 6.58 | 0.01* |
|  | Qc | 0.15 | 0.70 |
|  | QP | 0.72 | 0.39 |
| 135 | Pc | 0.00 | 0.99 |
|  | Qc | 5.09 | 0.02* |
|  | QP | 0.16 | 0.69 |

^a^ Pc, diet containing 0.5 mM *p*-coumaric acid; Qc, diet containing 0.25 mM quercetin; QP, diet containing 0.25 mM quercetin and 0.5 mM *p*-coumaric acid. (Total 5,400 bees were tested; *n* = 225 for each phytochemical sub-group; * *p* <0.05)
