# Supplementary material for: Biphasic concentration-dependent interaction between imidacloprid and dietary phytochemicals in honey bees (Apis mellifera)
Source: PLoS One. 2018 Nov 1;13(11):e0206625. doi: 10.1371/journal.pone.0206625 (PMC6211726; doi:10.1371/journal.pone.0206625)
Supplement: S4 Table — (DOCX) [file pone.0206625.s004.docx]

**S4 Table. Cox proportional hazards model analysis of effects of imidacloprid in different concentration with a phytochemical-free diet on adult bee longevity**

| Imidacloprid  (ppb) | Estimate | Standard error | *χ*^2^ | *df* | *P* | Hazard ratio |
| --- | --- | --- | --- | --- | --- | --- |
| 0 |  |  | 8.27 | 5.00 | 0.14 |  |
| 15^a^ | -0.06 | 0.09 | 0.38 | 1.00 | 0.54 | 0.94 |
| 45 | 0.08 | 0.10 | 0.71 | 1.00 | 0.40 | 1.09 |
| 75 | -0.08 | 0.10 | 0.68 | 1.00 | 0.41 | 0.92 |
| 105 | -0.17 | 0.10 | 3.36 | 1.00 | 0.07 | 0.84 |
| 135 | 0.00 | 0.09 | 0.00 | 1.00 | 0.99 | 1.00 |

^a^ Each sub-group has 225 tested bees.
